# Care for patients with hematologic neoplasms: contextual analysis resulting from a scoping review

**DOI:** 10.1590/0034-7167-2024-0533

**Published:** 2025-12-08

**Authors:** Isabelle Campos de Azevedo, Larissa Arielly Cunha da Silva, Estéfany Alves Augusto, Valéria Dantas de Azevedo, Viviane Euzébia Pereira Santos, Adriana Catarina de Souza Oliveira

**Affiliations:** I Universidade Federal do Rio Grande do Norte. Natal, Rio Grande do Norte, Brazil; II Universidad Católica de Murcia. Murcia, Spain

**Keywords:** Patients, Hematologic Neoplasms, Delivery of Health Care, Health Services, Nursing care., Pacientes, Neoplasias Hematológicas, Atención a la Salud, Servicios de Salud, Atención de Enfermeira.

## Abstract

**Objective:**

To analyze and map evidence in the literature on the contexts in which care for patients with hematologic neoplasms occurs within health services.

**Methods:**

This is a contextual analysis conducted in June 2024 through the development of a scoping review. The search for studies was carried out in national and international databases, as well as in thesis and dissertation portals, with the analysis focused on the immediate, specific, general, and metacontextual levels.

**Results:**

The sample, composed of thirty-two studies, revealed that the factors related to health care for patients with hematologic neoplasms in health services include: specific aspects of care; potentialities and barriers; actions that enhanced care; and the legislation that regulates it.

**Final Considerations:**

Complex interactions were observed, involving technical and interpersonal needs, as well as infrastructure-related challenges in the care of patients with neoplasms. These dynamics reveal important opportunities and obstacles within the care contexts.

## RESUMEN

**Objetivo:**

Analizar y mapear en la literatura evidencias sobre los contextos en los que ocurre la atención a pacientes con neoplasias hematológicas en los servicios de salud.

**Métodos:**

Se trata de un análisis contextual realizado en junio de 2024, a partir de la elaboración de una revisión de alcance (*scoping review*). La búsqueda de estudios se llevó a cabo en bases de datos nacionales e internacionales, así como en portales de tesis y disertaciones, con el análisis centrado en los contextos inmediato, específico, general y metacontexto.

**Resultados:**

La muestra, compuesta por treinta y dos estudios, evidenció que los factores relacionados con la atención en salud de pacientes con neoplasias hematológicas en los servicios de salud son: singularidades de la atención; potencialidades y barreras; acciones realizadas que impulsaron el cuidado; y las legislaciones que la regulan.

**Consideraciones Finales:**

Se observaron interacciones complejas que involucran necesidades técnicas, interpersonales y desafíos de infraestructura relacionados con la atención en salud de pacientes con neoplasias. Estas dinámicas revelan oportunidades y obstáculos importantes en los contextos de atención.

## INTRODUCTION

Hematologic neoplasms are characterized by the accumulation of mutations in bone marrow cells, referred to as leukemias or myelomas, or in the lymphatic system, referred to as lymphomas^(1)^. Due to the nature of onco-hematologic diseases and the aggressive and prolonged nature of their treatments, patients experience a wide range of symptoms that negatively impact various aspects of their physical, mental, and financial health^(2)^.

According to the World Health Organization (WHO), cancer is among the leading causes of death worldwide. Among these, hematologic neoplasms rank among the ten most commonly diagnosed types of cancer. In 2020, approximately 1,300,000 new cases of onco-hematologic diseases were reported globally^(3)^. In Brazil, the National Cancer Institute (INCA in Portuguese) projects an incidence rate of 14,170 new cases of hematologic neoplasms per 100,000 inhabitants for the period from 2023 to 2025^(4,5)^.

In this context, due to the high incidence and severity of these diseases, onco-hematologic patients require greater access to health services as they navigate the Health Care Networks (RAS in Portuguese) and utilize various medical technologies^(6)^, in addition to requiring care from a qualified multidisciplinary team, as evidenced by the clinical repercussions of hematologic malignancies^(7)^.

These patients should be supported by a range of healthcare professionals, including physicians, nurses, nutritionists, psychologists, dentists, and physical therapists, with the aim of providing holistic care that enhances the quality of clinical decision-making and service delivery^(7)^.

However, several factors undermine the healthcare provided to onco-hematologic patients, such as inadequate infrastructure, insufficient patient knowledge, sociocultural beliefs, and individual perceptions of health status^(8)^. These limitations negatively affect clinical prognosis and compromise both the effectiveness of treatment and patients’ quality of life^(9)^.

Therefore, understanding the contexts in which healthcare is delivered to patients with hematologic neoplasms can help identify the key factors related to this care. By analyzing the different care contexts, it is possible to identify challenges, optimize tools, improve service quality, and provide more effective, safe, and humanized care.

Given the above, the guiding research question was defined as: In what contexts does care for onco-hematologic patients occur within health services?

## OBJECTIVE

To analyze and map the evidence in the literature regarding the contexts in which care for patients with hematologic neoplasms occurs within health services.

## METHODS

### Ethical Aspects

It is important to note that, as this is a review study based on literature data sources, there was no involvement of human subjects. Therefore, approval by a Research Ethics Committee was not required.

### Theoretical-Methodological Framework

This is a contextual analysis study based on the framework proposed by Hinds, Chaves, and Cypress^(10)^, who define context as a set of four interactive layers that differ according to the expansion of a single fact or scenario. These layers are: immediate, specific, general, and metacontext. The immediate level focuses on the present and facilitates the identification of behavioral patterns in a given situation; the specific level refers to the immediate past and is influenced by the circumstances surrounding the event; the general level encompasses past and present interactions that may impact the phenomenon; and the metacontext operates between the past and present while shaping conditions for the future.

### Type of Study/Methodological Procedures

Prior to the literature search, a scoping review protocol was developed^(11,12)^ to guide the search process across data sources. This protocol was registered on the Open Science Framework (OSF) platform (10.17605/OSF.IO/SU8VG).

It is important to emphasize that the method proposed by the Joanna Briggs Institute was used solely to guide and structure the literature search in a systematic and organized manner. The methodological framework applied in this contextual analysis was that of Hinds, Chaves, and Cypress^(10)^.

This contextual analysis was developed in accordance with the criteria established by the COnsolidated criteria for REporting Qualitative research (COREQ) for qualitative studies^(13)^. To construct and analyze the contexts, a search was conducted in June 2024 across national and international databases, as well as gray literature, using the following keywords: 1) Hematologic Malignancies, 2) Health Care, 3) Patient Care Services. The search strategy employed Boolean operators “AND/OR” as follows: “Patients AND Hematologic neoplasms OR (hematologic malignancies) AND Delivery of health care OR (Health care) AND Health Services OR (Patient care services).”

Regarding the eligibility criteria, studies were included if they addressed the research question, met the objective of the study, and were fully available through the Federated Academic Community (CAFe) of the CAPES Journals Portal in electronic format. Editorials, letters to the editor, opinion articles, reflective and theoretical papers, abstracts, book chapters, and books were excluded.

No language or publication date restrictions were applied, and duplicate documents were considered only once.

Rayyan software^(14)^ was used to manage the collection and selection of studies. This process was conducted by a team of trained reviewers, working in pairs and independently. The selection process occurred in two stages: screening of titles and abstracts, followed by full-text reading of the selected studies for inclusion in the final sample.

The search was conducted in the following databases: PUBMED, SCOPUS, WEB OF SCIENCE, SCIENCE DIRECT, LILACS, COCHRANE, The National Library of Australia’s Trobe (TROVE), Academic Archive Online (DIVA), CAPES Theses and Dissertations Portal, Education Resources Information Center (ERIC), DART-Europe E-Theses Portal, Electronic Theses Online Service (EThOS), Open Access Scientific Repository of Portugal (RCAAP), National ETD Portal, and Latin American Theses and Dissertations. The search was conducted in pairs, and in the event of a disagreement, a third reviewer was consulted.

### Data Analysis

After selecting the references for analysis, all included materials were read in full, with particular attention given to elements characterizing each publication, including: database, language, year of publication, country, study objective, and the immediate, specific, general, and metacontext levels. The data were presented according to the contextual analysis proposed by Hinds, Chaves, and Cypress^(10)^, and grouped by context level, described, and visually represented to establish relationships among them.

Accordingly, in this study, the immediate context refers to the unique aspects of healthcare for patients with hematologic neoplasms within health services; the specific context addresses the strengths and barriers in delivering such care; the general context includes the actions implemented to strengthen this care; and the metacontext refers to elements related to laws and regulations in Brazil.

## RESULTS

**Figure 1:**
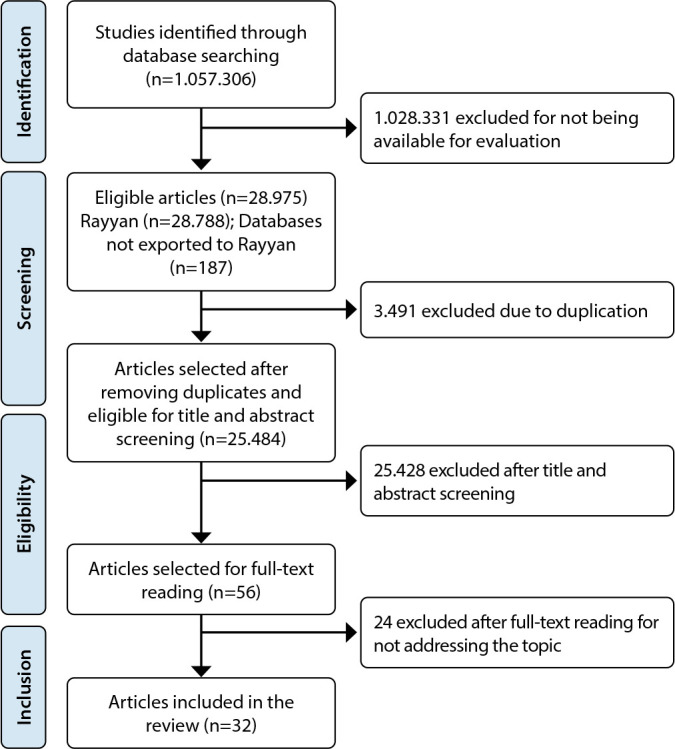
Study selection flowchart, 2024

In the selection of publications, 1,057,306 studies were identified, of which only 32 comprised the final sample (Figure 1).

The final sample of this study consisted of 32 publications released between 2009 and 2024. Of these, nine (28.12%) were published in 2023, six (18.75%) in 2022, four (12.50%) in 2020, three (9.40%) in 2021, two (6.25%) in each of the years 2018, 2019, and 2024, and one study (3.12%) in each of the years 2009, 2011, 2015, and 2017. Regarding the type of study, all were published as journal articles (100%).

Concerning the contextual levels proposed by Hinds, Chaves, and Cypress^(10)^, the immediate context identified the specific characteristics of healthcare for patients with hematologic neoplasms within health services. These include, for example, advance care planning, psychosocial support, increased access to services, dental evaluation prior to transplantation, training programs in health care, the use of quality indicators, specialized palliative care consultations, therapeutic education for self-care, rehabilitation programs involving physical exercise, laser therapy, and complementary and alternative medicine.

In relation to the specific context, both strengths and barriers in healthcare delivery to patients with hematologic neoplasms were identified. Among the strengths are: the use of health technologies to provide comfort, relieve concerns, and meet psychoeducational and informational needs, as well as to reduce hospital length of stay; the implementation of palliative care to increase educational opportunities for patients; the role of the interdisciplinary team; physical and emotional improvement with pain reduction; and patient self-management, including hygiene and care routines.

Regarding the barriers to care, notable issues include patient discomfort and lack of knowledge about certain procedures, infrastructure and support limitations, distance to healthcare services, ineffective communication, and lack of professional specialization in the field.

In the general context, key actions that enhanced care for patients with hematologic neoplasms within health services were highlighted, including the use of health technologies, the optimization of team knowledge and experience, the involvement of specialized teams, and the application of supportive care and symptom management strategies.

With respect to the metacontext-which encompasses legislation related to the healthcare of patients with hematologic neoplasms-prominent examples include the Mucositis Guidelines, the Patient Protection and Affordable Care Act, and the Clinical Practice Guidelines of the National Comprehensive Cancer Network (NCCN) in Oncology.

**Figure 2:**
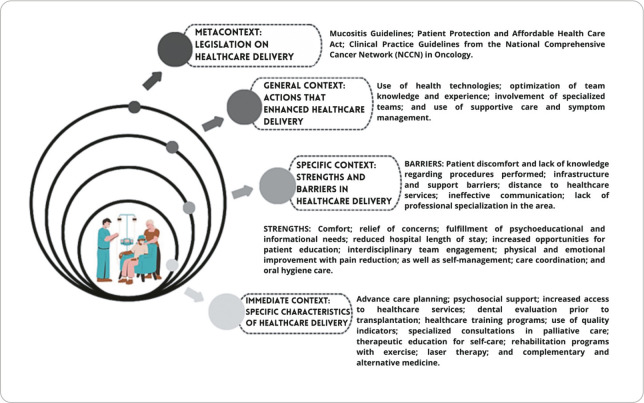
Contexts of healthcare for onco-hematologic patients within health services

The characterization of each contextual layer of healthcare for patients with hematologic neoplasms within health services is illustrated in Figure 2.

## DISCUSSION

Most of the studies in the final sample, being from recent years, indicate that the topic in question is still underexplored and not widely disseminated in the development of research on onco-hematologic patients, given the complexity they face in health services with regard to diagnosis and treatment^(6)^.

Anchored in the results obtained at each contextual level, the following subtopics present the findings of this study to facilitate the understanding of healthcare for patients with hematologic neoplasms within health services.

### Immediate Context - Specific characteristics of healthcare for patients with hematologic neoplasms within health services

Among the specific characteristics identified, advance care planning emerges as a strategy that empowers patients to understand and communicate their personal values and preferences, aiming for decision-making that is more aligned with their expectations regarding medical treatment. However, despite its potential to improve service quality, its use is infrequent in the care of onco-hematologic patients due to factors such as the lack of formal training among healthcare teams, which negatively impacts the quality of care provided^(15)^.

In this regard, professionals working in these services require specific technical skills due to the inherent demands of hematologic malignancies, which are often unmet and compromise the quality of care. They also need relational competencies to effectively engage with patients, family members/caregivers, and other members of the multidisciplinary team^(16)^.

The nurse-patient-caregiver-health team relationship goes beyond predefined roles and tasks. During moments of care, it is possible to understand the individual context and needs of each patient through health assessments, thereby identifying needs that are often overlooked-such as psychosocial support. This support can be provided by the entire healthcare team and also by family members or caregivers, aiming to improve social adaptation, quality of life, and potentially influence survival rates among onco-hematologic patients^(9,17)^.

Furthermore, oncological evaluation before Hematopoietic Stem Cell Transplantation (HSCT) is essential, as the treatment may lead to adverse systemic reactions, such as hemorrhages and infections, with the digestive system being the primary source of sepsis^(18)^. Among these infections, oral mucositis particularly affects pediatric onco-hematologic patients, compromising swallowing and treatment tolerance. Preventive strategies can be implemented to reduce the incidence of oral mucositis, such as high-power laser therapy, which has shown effective results in reducing pain and the severity of lesions^(19)^.

In this context, tools such as Complementary and Alternative Medicine (CAM) help relieve cancer-related symptoms and treatment toxicity. Through mind-body interventions, CAM is well accepted by patients for its ability to reduce stress and anxiety levels^(20)^.

On the other hand, a portion of individuals with hematologic neoplasms do not have their clinical needs adequately met. One contributing factor is the difficulty in accessing healthcare services for patients living far from treatment centers^(21)^.

As a result, care becomes weakened, and one strategy that can be implemented is empowering patients in health self-care, such as symptom self-management. Self-management, in collaboration with the medical team, promises active patient participation and a reduction in hospital admissions and costs. However, it proves limited when patients have doubts about their understanding of the condition or when the disease’s pathophysiology presents unpredictability^(22)^.

Care for patients with hematologic neoplasms within health services is broad and includes palliative care, which aims to improve the quality of life for those facing prognostic uncertainty regarding treatment. However, studies show that patients have less frequent and more delayed access to specialized palliative care consultations. This is due to factors such as a lack of clarity about what constitutes advanced disease in hematology and the difficulty in identifying patients who require palliative care^(23)^.

In this regard, the use of quality indicators associated with cancer supportive care is a strategy to standardize care delivery. However, its implementation is not always effective. Furthermore, developing quality indicators requires professional training and appropriate systems, which may be hindered by gaps in public funding^(24)^.

### Specific Context - Strengths and barriers in healthcare for patients with hematologic neoplasms within health services

Among the actions identified as enhancing healthcare for onco-hematologic patients are comfort, relief of concerns, fulfillment of psychoeducational and informational needs, reduced hospital length of stay, increased opportunities for patient education, interdisciplinary team involvement, physical and emotional improvement with pain reduction, as well as self-management, care coordination, and oral hygiene care.

In the context of care, the role of the dentist on the interdisciplinary team is noteworthy, since a significant number of patients with hematologic neoplasms require interventions involving the oral cavity. Additionally, professional management includes continuous monitoring and the provision of specific oral care guidelines tailored to each patient’s individual needs^(18)^.

Through the health education process, users of onco-hematologic services are encouraged to take an active role in managing their own care, aiming to reduce the number of hospital admissions. Moreover, patient autonomy is essential, as individuals who feel empowered and well-informed make more conscious and appropriate decisions. Consequently, this improves treatment effectiveness and increases both satisfaction and quality of life^(25,26)^.

In the context of palliative care, such autonomy becomes even more critical, as it seeks to provide symptom relief and reduce suffering in patients with the most severe forms of hematologic neoplasms. In this way, active participation in care management enables a more personalized approach aligned with the patient’s values and preferences, resulting in more humanized and effective care^(27)^.

In this regard, early palliative care services have shown positive outcomes in reducing the number of patients reporting moderate to severe pain. Furthermore, physical and emotional improvement is achieved through the involvement of various specialties. As a result, patient comfort is significantly enhanced, providing a better quality of life during treatment. Therefore, the focus on pain relief and emotional well-being-through a multidisciplinary approach-ensures that patients receive holistic care aimed not only at disease control but also at comfort and dignity^(27)^.

However, the identified barriers included patient discomfort and a lack of knowledge about the procedures performed; infrastructure and support limitations; distance from healthcare services; limited communication; and a lack of professional specialization in the field. Advance care planning involves discussions between healthcare professionals and patients involved in the care process and allows for shared decision-making regarding the goals of care^(28)^.

A study^(15)^ shows that the timing of such planning is individualized and varies for each patient, with earlier discussions taking place in cases with poorer prognoses. In this context, patient discomfort during the oncologist’s explanation of care plans and guidance is noteworthy, as is their lack of understanding of the procedures involved-both of which contribute to anxiety about future treatment.

Furthermore, communication difficulties between patients and family members regarding hematologic neoplasms are often exacerbated by challenges in expressing emotions and by the fact that the subject is not widely discussed. As a result, patients and families may find it difficult to speak openly about the emotional impact of the disease due to fear or concern about burdening others. These unspoken emotions can create an environment of limited communication, where genuine concerns and needs remain unaddressed. Additionally, the sensitive and often stigmatized nature of hematologic neoplasms can lead to uncomfortable silences, making it difficult to discuss the patient’s condition and the implications of treatment^(9)^.

In line with the above, the treatment of onco-hematologic patients may present additional barriers, such as those related to infrastructure and support. A study^(21)^ conducted with staff from cancer centers designated by the National Cancer Institute found that 45% of the team believed that most of the survivorship needs of patients with blood cancer who had completed treatment were not adequately met by their center or by other organizations. Among the reported barriers were the distance between patients’ homes and treatment services, as well as a lack of physician adherence to new practices.

From this perspective, in onco-hematologic services, the importance of having specialized professionals with appropriate training and qualifications in new models of care is emphasized. This approach enhances interdisciplinary collaboration within the team and also increases the engagement of the hematology palliative care team, resulting in more effective strategies and approaches by the professionals involved^(23)^.

### General Context - Actions that have enhanced healthcare for patients with hematologic neoplasms within health services

The use of technology has brought innovations to the healthcare field, as evidenced by the implementation of tools such as guidelines, protocols, and mobile applications. Through these strategies, users of onco-hematologic services develop skills related to self-care and symptom self-management, enabling more active participation in their treatment and decision-making processes^(25,29)^.

Moreover, health technologies offer essential support, particularly for patients who have undergone hematopoietic stem cell transplantation (HSCT), due to the need for specialized care. For example, a study showed that women who underwent HSCT experienced fertility-related changes, and as an alternative to manage these complications, a practical guide for gynecological and reproductive health was developed. This tool assists in reproductive health counseling and has proven effective in optimizing the knowledge and experience of the healthcare team^(29)^.

In this context, the involvement of specialized teams is crucial due to the multisystemic repercussions of hematologic malignancies. Therefore, healthcare teams must work collaboratively to improve the quality of care provided. Interdisciplinary collaboration enables a comprehensive and personalized approach, enriching collective knowledge and promoting more effective practices. As a result, there is an improvement in the quality of care and in the early identification of complications, which positively impacts the quality of life of onco-hematologic patients. After hospital discharge, patient autonomy is reinforced through clear guidance, continuous support, and the use of health tools, empowering them to manage their health more independently^(30)^.

From this perspective, mobile health (mHealth) applications are tools that help reduce hospital length of stay by allowing continuous monitoring of patients, including real-time tracking of vital signs and treatment progress. Additionally, they provide medication reminders, appointment scheduling, and facilitate treatment adherence and complication prevention. These tools also enhance communication between patients and healthcare professionals, reduce the need for hospital visits, and promote patient autonomy-resulting in better clinical outcomes and greater satisfaction^(26)^.

Metacontext - Legislation on healthcare for patients with hematologic neoplasms within health services

The creation and implementation of laws, policies, guidelines, and ordinances aimed at optimizing healthcare for onco-hematologic patients are observed in various contexts. For example, the Patient Protection and Affordable Care Act (PPACA), enacted in 2010 in the United States, aims to increase access to healthcare and reduce service costs. This legislation was necessary because a significant portion of the U.S. population lacked health insurance coverage, and there was no national healthcare system in place, such as the Unified Health System (SUS) in Brazil^(30)^.

In addition, nonprofit institutions have been established to improve and facilitate care for cancer patients and enhance their quality of life. Accordingly, the guidelines developed by the National Comprehensive Cancer Network (NCCN), established in 2022, provide practical recommendations to minimize and treat infections in cancer patients, particularly due to the frequent occurrence of febrile neutropenia in patients with blood cancers. The NCCN plays a crucial role in standardizing and improving care for cancer patients, including those with hematologic neoplasms, by offering effective and innovative treatments and promoting a better quality of life^(31)^.

Another example is the Multinational Association of Supportive Care in Cancer / International Society of Oral Oncology (MASCC/ISOO), which in 2019 issued updated recommendations for the prevention and treatment of oral mucositis in cancer patients. This condition frequently occurs as a side effect of treatments such as chemotherapy and radiotherapy. In this context, strategies to reduce the incidence and severity of mucositis are essential to clinical management, including appropriate oral care, pain management techniques, and pharmacological interventions. The emphasis on a multidisciplinary approach aims to improve patients’ quality of life and optimize outcomes from oncological treatments^(32)^.

### Study Limitations

The potential limitations of this study include the inclusion of only studies that were publicly available in full text, which may have resulted in the exclusion of relevant studies that did not meet this criterion. Additionally, the publications selected for the sample generally had low levels of evidence (levels III and IV), with noted methodological weaknesses.

### Contributions to Nursing, Health, or Public Policy

This study contributes to understanding the complexities involved in providing care to patients with hematologic neoplasms, while also identifying areas in need of improvement, such as ongoing professional training and expanded access to healthcare services. Therefore, future research should focus on developing strategies to overcome the identified barriers and exploring innovative technologies and approaches that can optimize care for these patients.

## FINAL CONSIDERATIONS

Regarding the factors identified in the contexts analyzed in this study on care for onco-hematologic patients within health services, complex interactions were observed-ranging from technical and interpersonal needs to challenges related to infrastructure and communication.

Among the main findings, the importance of advance care planning, the need for continuous professional training, and the use of health technologies to enhance the quality of care are emphasized. However, the lack of formal training among healthcare teams and the difficulties in accessing services for patients in remote areas were identified as factors that may compromise the effectiveness of care.

Thus, it is relevant to conduct and disseminate further research on this topic, grounded in practical care experiences, and to develop strategies to strengthen healthcare delivery for onco-hematologic patients.

## Data Availability

Data usage not reported, research data not used.
