# Supplementary material for: Care for patients with hematologic neoplasms: contextual analysis resulting from a scoping review
Source: Rev Bras Enferm. 2025 Dec 8;78(5):e20240533. doi: 10.1590/0034-7167-2024-0533 (PMC12707764; doi:10.1590/0034-7167-2024-0533)
Supplement: Translation

# Translation: Assistência a pacientes com neoplasias hematológicas: análise contextual resultante de uma revisão de escopo

**DOI:** 10.1590/0034-7167-2024-0533pt

**Authors:** Isabelle Campos de Azevedo, Larissa Arielly Cunha da Silva, Estéfany Alves Augusto, Valéria Dantas de Azevedo, Viviane Euzébia Pereira Santos, Adriana Catarina de Souza Oliveira

**Affiliations:** I Universidade Federal do Rio Grande do Norte. Natal, Rio Grande do Norte, Brasil; II Universidad Católica de Murcia. Murcia, España

**Keywords:** Pacientes, Neoplasias Hematológicas, Atenção à, Saúde, Serviços de Saúde, Cuidados de Enfermagem.

## Abstract

**Objetivo:**

Analisar e mapear na literatura evidências de em quais contextos ocorre a assistência a pacientes com neoplasias hematológicas nos serviços de saúde.

**Métodos:**

Consiste em uma análise de contexto realizada em junho de 2024, mediante elaboração de uma *scoping review*. A busca por estudos deu-se em bases de dados nacionais e internacionais, além de portais de teses e dissertações, com análise centrada nos contextos imediato, específico, geral e metacontexto.

**Resultados:**

A amostra, composta por trinta e dois estudos, evidenciou que os fatores relacionados à assistência à saúde de pacientes com neoplasias hematológicas nos serviços de saúde são: singularidades da assistência; potencialidades e entraves; ações realizadas que impulsionaram o cuidado; e as legislações que a regulamentam.

**Considerações Finais:**

Observou-se interações complexas que envolvem necessidades técnicas, interpessoais e desafios de infraestrutura relacionados à assistência à saúde de pacientes com neoplasias. Essas dinâmicas revelam oportunidades e obstáculos importantes nos contextos de atendimento.

## INTRODUÇÃO

As neoplasias hematológicas são caracterizadas pelo acúmulo de mutações de células na medula óssea, denominadas leucemias ou mielomas, ou no sistema linfático, chamados de linfomas^(1)^. Devido às características das doenças onco-hematológicas e aos tratamentos agressivos e prolongados, os pacientes sofrem com uma grande quantidade de sintomas, que impactam negativamente várias áreas de sua saúde física, mental e financeira^(2)^.

Segundo a Organização Mundial da Saúde (OMS), o câncer está entre as principais causas de morte no mundo. Dentre estes, as neoplasias hematológicas estão entre os 10 tipos de câncer mais comumente diagnosticados. Em 2020, aproximadamente 1.300.000 novos casos de doenças onco-hematológicas foram constatados mundialmente^(3)^. Já no Brasil, o Instituto Nacional de Câncer (INCA) projeta uma taxa de incidência de 14.170 novos casos de neoplasias hematológicas a cada 100 mil habitantes no período de 2023 a 2025^(4,5)^.

Nessa perspectiva, devido à alta incidência e gravidade dessas doenças, os pacientes onco-hematológicos necessitam de maior acesso aos serviços de saúde, ao percorrerem as Redes de Assistência à Saúde (RAS) e utilizarem as diversas tecnologias médicas^(6)^, além da necessidade de cuidados por uma equipe multiprofissional capacitada, evidenciada pelas repercussões clínicas das malignidades hematológicas^(7)^.

Esses pacientes devem ser atendidos por uma variedade de profissionais de saúde, dentre eles, médicos, enfermeiros, nutricionistas, psicólogos, dentistas e fisioterapeutas, com o objetivo de fornecerem cuidados holísticos para uma melhor qualidade do processo de tomada de decisão e atendimento clínico^(7)^.

Entretanto, existem fatores que fragilizam a assistência à saúde do paciente onco-hematológico, como infraestrutura inadequada, conhecimento insuficiente do usuário, crenças socioculturais e a percepção individual do estado de saúde^(8)^. Essas limitações influenciam negativamente o prognóstico clínico e comprometem a eficácia do tratamento e a qualidade de vida dos pacientes^(9)^.

Por conseguinte, compreender em quais contextos ocorre a assistência à saúde de pacientes com neoplasias hematológicas nos serviços de saúde pode contribuir para identificar os principais fatores relacionados a esse cuidado. Ao analisar os diferentes contextos de assistência dos pacientes, é possível identificar os desafios, otimizar ferramentas, melhorar a qualidade dos serviços e proporcionar um cuidado mais eficaz, seguro e humanizado.

Diante do exposto, definiu-se como questão norteadora: Em quais contextos ocorre a assistência de pacientes onco-hematológicos nos serviços de saúde?

## OBJETIVO

Analisar e mapear na literatura evidências de em quais contextos ocorre a assistência de pacientes com neoplasias hematológicas nos serviços de saúde.

## MÉTODOS

### Aspectos éticos

Destaca-se que, por se tratar de um estudo de revisão em fontes de dados da literatura, não houve envolvimento de seres humanos, o que dispensa a aprovação por parte de um Comitê de Ética em Pesquisa.

### Referencial teórico-metodológico

Este é um estudo de análise de contexto fundamentado no referencial de Hinds, Chaves e Cypress^(10)^, que definem o contexto como um conjunto de quatro camadas interativas que se diferenciam pela expansão de um mesmo fato ou cenário. São elas: contexto imediato, específico, geral e metacontexto. O imediato tem foco no presente, facilitando a identificação de padrões de comportamento de uma situação; o específico concentra-se no passado imediato e é influenciado pelas circunstâncias que ocorreram no evento; o geral compreende interações passadas e atuais que terão impactos no fenômeno; e o metacontexto opera entre passado e presente, além de moldar condições para o futuro.

### Tipo de estudo/Procedimentos metodológicos

Previamente à investigação na literatura, foi elaborado um protocolo de revisão de escopo^(11,12)^, com o objetivo de nortear o processo de busca nas fontes de dados, o qual se encontra registrado na plataforma de estudos Open Science Framework (OSF) (10.17605/OSF.IO/SU8VG). É importante destacar que o método proposto pelo Joanna Briggs Institute foi utilizado apenas para nortear e orientar a busca por estudos na literatura de forma metódica e estruturada. O referencial metodológico utilizado para o desenvolvimento da presente análise de contexto foi o de Hinds, Chaves e Cypress^(10)^.

Esta análise contextual foi construída com base nos critérios determinados pelo *COnsolidated criteria for REporting Qualitative research* (COREQ) para pesquisas qualitativas^(13)^. Para a construção e análise dos contextos, realizou-se uma busca em junho de 2024 em bases de dados nacionais e internacionais, como também na literatura cinzenta, a partir das palavras-chave: 1) Malignidades hematológicas/*Hematologic Malignancies*, 2) Cuidados de saúde/*Helth Care*, 3) Serviços de atenção ao paciente/*Patient Care Services*. Foi adotada a estratégia de busca, mediante os operadores boleandos “AND/OR”: “*Patients* AND *Hematologic neoplasms* OR (*hematologic malignancies*) AND *Delivery of health care* OR (Health care) AND *Health Services* OR (*Patient care services*)”.

Com relação aos critérios de elegibilidade, foram incluídas publicações que respondessem à questão de pesquisa, atendessem ao objetivo do estudo e estivessem disponíveis na íntegra por meio da Comunidade Acadêmica Federada (CAFe) do Periódicos CAPES, em meio eletrônico. Foram excluídos os estudos em formatos de editoriais, cartas ao editor, artigos de opinião, reflexivos e teóricos, resumos, capítulos de livro e livros. Não foram impostas restrições de idioma ou limite temporal, e os documentos duplicados foram considerados apenas uma vez.

Utilizou-se o software Rayyan^(14)^ para o gerenciamento da coleta e seleção dos estudos, e esse processo foi realizado por uma equipe de revisores treinados, em pares e de forma independente. O processo de seleção ocorreu em duas etapas: leitura do título e resumo, seguida pela leitura na íntegra dos estudos selecionados para compor a amostra.

A coleta ocorreu nas bases de dados PUBMED, SCOPUS, WEB OF SCIENCE, SCIENCE DIRECT, LILACS, COCHRANE, The National Library of Australia’s Trobe (TROVE), Academic Archive Online (DIVA), Portal de Teses e Dissertações da CAPES, *Education Resources Information Center (ERIC), DART-Europe E-Theses Portal, Electronic Theses Online Service (EThOS)*, Repositório Científico de Acesso Aberto de Portugal (RCAAP), *National ETD Portal* e Teses e Dissertações da América Latina. A busca foi realizada aos pares e, em caso de divergência entre eles, um terceiro revisor era consultado.

### Análise dos dados

Após a definição das referências para análise, procedeu-se à leitura integral de todo o material selecionado, com destaque para os elementos que caracterizam cada publicação, dentre eles: base de dados, idioma, ano de publicação, país, objetivo do estudo e os contextos específico, imediato, geral e metacontexto. Os dados foram apresentados conforme a análise de contexto proposta por Hinds, Chaves e Cypress^(10)^, e agrupados de acordo com cada nível de contexto, descritos e representados por meio de imagem, com o propósito de estabelecer relações entre eles. Dessa forma, para o presente estudo, o contexto imediato será as singularidades da assistência à saúde de pacientes com neoplasias hematológicas nos serviços de saúde; o específico abordará as potencialidades e os entraves da assistência à saúde desses pacientes; o contexto geral será as ações realizadas que impulsionaram essa assistência; e o metacontexto fará referência aos elementos relacionados às leis e regulamentações no Brasil.

## RESULTADOS

Na seleção das publicações, foram identificados 1.057.306 estudos, dos quais apenas 32 constituíram a amostra final (Figura 1).

**Figura 1:** Fluxograma de seleção dos estudos, 2024

A amostra final deste estudo consistiu em 32 materiais publicados no período de 2009 a 2024. Destes, nove (28,12%) em 2023, seis (18,75%) em 2022, quatro (12,50%) em 2020, três (9,40%) em 2021, dois (6,25%) nos anos de 2018, 2019 e 2024, e um (3,12%) estudo publicado nos anos de 2009, 2011, 2015 e 2017. Acerca do tipo de estudo, todos estavam em formato de artigo (100%).

No que concerne aos níveis contextuais propostos por Hinds, Chaves e Cypress^(10)^, identificou-se no contexto imediato as singularidades da assistência à saúde de pacientes com neoplasias hematológicas nos serviços de saúde, como, por exemplo, o planejamento antecipado de cuidados, apoio psicossocial, aumento do acesso aos serviços, avaliação odontológica antes da realização do transplante, programa de capacitação em cuidados de saúde, utilização de indicadores de qualidade, consulta especializada em cuidados paliativos, educação terapêutica para o autocuidado, programas de reabilitação com uso de exercícios, laserterapia e medicina complementar e alternativa.

Em relação ao contexto específico, evidenciaram-se as potencialidades e os entraves da assistência à saúde de pacientes com neoplasias hematológicas nos serviços de saúde, dentre os quais vale ressaltar, como potencialidades: uso de tecnologias em saúde para proporcionar conforto, alívio de preocupações, satisfação das necessidades psicoeducacionais e de informações, além de proporcionar menor tempo de internação hospitalar; a realização de cuidados paliativos para favorecer o aumento de oportunidades de educação do paciente; a atuação da equipe interdisciplinar; melhora física e emocional com diminuição da dor; além da autogestão, gestão e cuidados com a higiene oral.

No que diz respeito aos entraves dos cuidados, destacam-se o desconforto e a falta de conhecimento do paciente sobre determinados procedimentos realizados, barreiras de infraestrutura e apoio, distância para acesso aos serviços, falta de comunicação eficaz e falta de especialização dos profissionais na área.

No contexto geral, destacam-se as ações realizadas que impulsionaram a assistência à saúde de pacientes com neoplasias hematológicas nos serviços de saúde, dentre elas, o uso de tecnologias em saúde, a otimização do conhecimento e da experiência da equipe, o envolvimento da equipe especializada e o uso de cuidados de suporte e manejo dos sintomas.

No tocante ao metacontexto, que engloba as legislações sobre a assistência à saúde de pacientes com neoplasias hematológicas nos serviços de saúde, destacam-se as Diretrizes de Mucosite, a Lei de Proteção ao Paciente e Cuidados de Saúde Acessíveis e as Diretrizes de Prática Clínica da *National Comprehensive Cancer Network* (NCCN) em Oncologia.

A caracterização de cada camada contextual da assistência à saúde de pacientes com neoplasias hematológicas nos serviços de saúde encontra-se ilustrada na Figura 2.

**Figura 2:** Contextos da assistência à saúde de pacientes onco-hematológicos nos serviços de saúde

## DISCUSSÃO

A maioria dos estudos da amostra final, por serem de anos recentes, mostra que o tema em questão ainda é pouco discutido e difundido no desenvolvimento de estudos acerca de pacientes onco-hematológicos, haja vista a complexidade que enfrentam nos serviços de saúde com relação ao diagnóstico e tratamento^(6)^.

Ancorado nos resultados obtidos em cada camada contextual, os subtemas a seguir apresentam os achados deste estudo, de modo a facilitar a compreensão da assistência à saúde de pacientes com neoplasias hematológicas nos serviços de saúde.

### Contexto imediato - Singularidades da assistência à saúde de pacientes com neoplasias hematológicas nos serviços de saúde

Dentre as singularidades apresentadas, o planejamento antecipado de cuidados surge como uma estratégia que capacita os pacientes na compreensão e na comunicação de seus valores e preferências pessoais, com o objetivo de tomadas de decisões mais alinhadas com suas expectativas em relação ao tratamento médico. Entretanto, apesar de apresentar um potencial na qualidade do serviço, seu uso não é frequente na assistência aos pacientes onco-hematológicos, devido a fatores como a falta de treinamento formal da equipe de saúde, que influenciam negativamente no atendimento^(15)^.

Nesse sentido, os profissionais que atuam nesses serviços necessitam de habilidades técnicas específicas, devido às demandas inerentes às malignidades hematológicas, que muitas vezes não são atendidas e comprometem a qualidade do cuidado prestado, além do desenvolvimento de capacidade relacional com o paciente, familiares/cuidadores e com os demais membros da equipe multiprofissional^(16)^.

A relação enfermeiro-paciente-cuidador-equipe de saúde vai além das ações e funções pré-estabelecidas pelos serviços. Durante os momentos de cuidado, é possível conhecer os contextos e especificidades de cada paciente por meio da avaliação em saúde e, com isso, identificar necessidades que muitas vezes ficam em segundo plano ou não são identificadas, a exemplo do apoio psicossocial, que pode ser fornecido por toda a equipe e também por familiares/cuidadores, com a finalidade de melhorar a adaptação social, a qualidade de vida e influenciar na taxa de sobrevida dos pacientes onco-hematológicos^(9,17)^.

Ademais, a avaliação oncológica antes do Transplante de Células-Tronco Hematopoéticas (TCTH) é importante, pois o tratamento pode ocasionar reações sistêmicas adversas, como hemorragias e infecções, sendo o sistema digestório a principal fonte de sepse^(18)^. Dentre essas infecções, a mucosite oral afeta pacientes onco-hematológicos, principalmente na pediatria, comprometendo a deglutição e a tolerância ao tratamento. Para isso, estratégias de prevenção podem ser implementadas para reduzir a incidência da mucosite oral, como a terapia a laser de alta potência, que demonstra resultados eficazes em relação à dor e à gravidade das lesões^(19)^.

Nessa perspectiva, ferramentas como a Medicina Complementar e Alternativa (MCA) auxiliam no alívio dos sintomas do câncer e da toxicidade do tratamento. Assim, por meio das intervenções mente-corpo, a MCA é bem aceita pelos pacientes por sua capacidade de reduzir os níveis de estresse e ansiedade^(20)^.

Em contrapartida, uma parcela dos indivíduos com neoplasias hematológicas não tem suas necessidades clínicas atendidas adequadamente. Um dos fatores é a dificuldade de acesso aos serviços de saúde para os pacientes que residem longe do centro onde realizam o tratamento^(21)^. Como resultado, há uma fragilização na assistência prestada, e uma das estratégias que podem ser implementadas é a capacitação dos pacientes para o autocuidado em saúde, como o autogerenciamento dos sintomas. A autogestão, em conjunto com a equipe médica, é uma promessa de participação ativa, com redução de internações e custos. Contudo, demonstra-se limitada quando o paciente apresenta incertezas em relação à compreensão ou quando a fisiopatologia da doença se mostra imprevisível^(22)^.

A assistência a pacientes com neoplasias hematológicas nos serviços de saúde é ampla e se estende aos cuidados paliativos, que buscam melhorar a qualidade de vida daqueles que estão em situação de imprevisibilidade prognóstica em relação ao tratamento. Entretanto, estudos demonstram que os pacientes têm acesso menos frequente e mais tardio às consultas especializadas em cuidados paliativos. Isso se deve a fatores como a falta de clareza sobre o que constitui uma doença avançada na hematologia e à dificuldade na identificação de pacientes que necessitam de cuidados paliativos^(23)^.

Para isso, a utilização de indicadores de qualidade associados à prestação de cuidados de suporte ao câncer é uma estratégia para a padronização da assistência. Entretanto, sua implementação nem sempre é eficaz. Ademais, o desenvolvimento de indicadores de qualidade requer a capacitação dos profissionais e sistemas apropriados, o que pode representar uma dificuldade devido a lacunas no financiamento público^(24)^.

### Contexto Específico - Potencialidades e entraves da assistência à saúde de pacientes com neoplasias hematológicas nos serviços de saúde

Dentre as ações elencadas que potencializam a assistência à saúde para pacientes onco-hematológicos, destacam-se: o conforto, o alívio de preocupações, a satisfação das necessidades psicoeducacionais e de informações, menor tempo de internação hospitalar, aumento de oportunidades de educação do paciente, atuação da equipe interdisciplinar, melhora física e emocional com diminuição da dor, além da autogestão, gestão e cuidados com a higiene oral. No contexto da assistência, ressalta-se a presença do odontólogo na equipe interdisciplinar, uma vez que grande parte dos pacientes com neoplasias hematológicas demanda intervenções na cavidade oral. Além disso, a gestão profissional envolve o monitoramento contínuo e o fornecimento de orientações específicas sobre cuidados orais, ajustadas às necessidades individuais dos pacientes^(18)^.

Por meio do processo de educação em saúde, o usuário dos serviços onco-hematológicos é incentivado a atuar de maneira autônoma na gestão do seu cuidado, com vistas à redução do número de internações hospitalares. Além disso, a autonomia do paciente é essencial, pois, ao se sentir capacitado e melhor informado, o indivíduo toma decisões mais conscientes e adequadas. Consequentemente, isso melhora a eficácia do tratamento e aumenta a satisfação e a qualidade de vida^(25,26)^.

Em relação aos cuidados paliativos, essa autonomia é ainda mais crítica, pois visa proporcionar alívio de sintomas e sofrimento em pacientes com as formas mais graves de neoplasias hematológicas. Dessa forma, a participação ativa na gestão do cuidado permite uma abordagem mais personalizada e alinhada com os valores e desejos do paciente, resultando em um cuidado mais humanizado e eficiente^(27)^.

Nesse ínterim, o serviço de cuidados paliativos precoces apresenta resultados positivos em relação à diminuição dos pacientes que relataram dor moderada a intensa. Além disso, a melhora física e emocional é alcançada com a participação de várias especialidades. Como consequência, o conforto dos pacientes é significativamente aumentado, proporcionando uma melhor qualidade de vida durante o tratamento. Logo, o foco no alívio da dor e no bem-estar emocional, por meio de uma abordagem multidisciplinar, assegura que os pacientes recebam um cuidado holístico, voltado não apenas para o controle da doença, mas também para o seu conforto e dignidade^(27)^.

Contudo, os entraves elencados foram: o desconforto e a falta de conhecimento do paciente sobre os procedimentos realizados; barreiras de infraestrutura e apoio; distância para acesso aos serviços; comunicação limitada; e falta de especialização dos profissionais na área. O planejamento antecipado de cuidados envolve a discussão entre os profissionais e os pacientes envolvidos no cuidado e permite a tomada de decisão compartilhada quanto aos objetivos no processo de saúde^(28)^. Estudo^(15)^ mostra que o momento desse planejamento é individualizado e variável para cada paciente, com discussão mais precoce para os casos com pior prognóstico. Nisso, destaca-se o desconforto que os pacientes sentem durante o repasse de informações pelo médico oncologista sobre o planejamento e orientações de seus cuidados, bem como a falta de conhecimento sobre os procedimentos realizados, o que resulta em ansiedade quanto ao tratamento futuro.

Além disso, a dificuldade de comunicação entre pacientes e familiares sobre neoplasias hematológicas é frequentemente acentuada pela dificuldade em expressar sentimentos e pelo fato de que o assunto não é amplamente discutido. Portanto, pacientes e familiares podem achar desafiador falar abertamente sobre o impacto emocional da doença devido ao medo ou à preocupação em sobrecarregar os outros. Como resultado, essas emoções não expressas podem criar um ambiente de comunicação limitada, onde preocupações e necessidades reais permanecem não abordadas. Além disso, a natureza sensível e, muitas vezes, estigmatizada das neoplasias hematológicas pode levar a um silêncio constrangedor, dificultando a abertura sobre o estado do paciente e as implicações do tratamento^(9)^.

Em consonância com o exposto, o tratamento do paciente onco-hematológico pode apresentar outros tipos de barreiras, a exemplo da infraestrutura e apoio. Estudo^(21)^ realizado com a equipe nos Centros de Câncer designados pelo Instituto Nacional do Câncer aponta que 45% da equipe relataram acreditar que a maioria das necessidades de sobrevivência dos pacientes com câncer no sangue que concluíram o tratamento não foi adequadamente atendida por seu centro ou por outras organizações. Dentre essas barreiras, destacam-se a distância da casa dos pacientes para o acesso aos serviços e a falta de adesão do médico a novas práticas.

Nessa perspectiva, nos serviços onco-hematológicos, destaca-se a importância de profissionais especializados na área, com formação e capacitação adequadas às novas práticas de cuidado. Essa ação proporciona uma melhora na colaboração interdisciplinar da equipe, como também gera aumento na atividade da equipe de cuidados paliativos de hematologia, com melhor abordagem por parte dos profissionais envolvidos^(23)^.

### Contexto Geral - Ações realizadas que impulsionaram a assistência à saúde de pacientes com neoplasias hematológicas nos serviços de saúde

O uso de tecnologias trouxe inovações no âmbito da saúde, evidenciado pelo uso de ferramentas nos serviços, como guias, protocolos e aplicativos móveis. Logo, com o uso dessas estratégias, os usuários dos serviços onco-hematológicos desenvolvem habilidades em relação ao autocuidado e ao autogerenciamento dos sintomas, com participação ativa no seu tratamento e na tomada de decisões^(25,29)^.

Ademais, as tecnologias em saúde oferecem suporte principalmente a pacientes que foram submetidos ao TCTH, devido à necessidade de cuidado especializado. A título de exemplo, estudo desenvolvido mostra que mulheres submetidas ao TCTH sofrem com alterações na fertilidade e, como alternativa para gerenciar essas complicações, foi desenvolvido um guia prático para a saúde ginecológica e reprodutiva. Assim, a ferramenta auxilia no aconselhamento sobre a saúde reprodutiva, além de mostrar-se efetiva em relação à otimização do conhecimento e da experiência da equipe^(29)^.

Neste ínterim, o envolvimento da equipe especializada é crucial devido às repercussões multissistêmicas das malignidades hematológicas. Nesse sentido, é necessário que as equipes de saúde atuem em conjunto para melhorar o cuidado prestado aos pacientes. Além disso, a colaboração interdisciplinar permite uma abordagem abrangente e personalizada, de forma a enriquecer o conhecimento coletivo e promover práticas mais eficazes. Consequentemente, há uma melhora na qualidade do atendimento e na identificação de complicações, o que impacta positivamente a qualidade de vida dos pacientes onco-hematológicos. Após a alta, a autonomia do paciente é reforçada por meio de orientações claras, apoio contínuo e uso de ferramentas em saúde, capacitando-o a gerenciar sua saúde de forma mais independente^(30)^.

Nesta perspectiva, serviços de aplicativos móveis em saúde (mHealth) são ferramentas que reduzem o tempo de internação hospitalar ao permitir o monitoramento contínuo dos pacientes, com acompanhamento em tempo real dos sinais vitais e progresso do tratamento. Além disso, fornecem lembretes para medicação, acesso a consultas e facilitam a adesão ao tratamento e a prevenção de complicações. Assim, melhoram a comunicação entre pacientes e profissionais, com a redução de visitas hospitalares e promoção da autonomia dos pacientes, resultando em melhores desfechos clínicos e maior satisfação^(26)^.

### Metacontexto - Legislações acerca da assistência à saúde de pacientes com neoplasias hematológicas nos serviços de saúde

A criação e execução das leis, políticas, diretrizes e portarias instituídas com o objetivo de otimizar a assistência à saúde de pacientes onco-hematológicos é observada em amplos contextos. A título de exemplo, tem-se a Lei de Proteção e Cuidado Acessível ao Paciente (*Patient Protection and Affordable Care Act* - PPACA), promulgada em 2010 pela sociedade americana, que tem por objetivo aumentar o acesso aos cuidados de saúde e reduzir os custos dos serviços. Isso se deve ao fato de que, nos Estados Unidos, boa parte da população não tinha cobertura por plano de saúde, além de não existir um serviço nacional, como, por exemplo, o Sistema Único de Saúde (SUS) no Brasil^(30)^.

Concatenado a isso, são criadas instituições sem fins lucrativos para melhorar e facilitar o atendimento de pacientes com câncer e elevar a qualidade de vida. Com isso, diretrizes como as da *National Comprehensive Cancer Network* (NCCN), instituídas em 2022, visam fornecer orientações práticas para minimizar e tratar infecções em pacientes com câncer, devido à recorrência de neutropenia febril em pacientes com neoplasias sanguíneas. Além disso, a NCCN desempenha um papel crucial na padronização e melhoria da assistência a pacientes com câncer, incluindo as neoplasias hematológicas, de forma a proporcionar tratamentos eficazes e inovadores, além de uma melhor qualidade de vida^(31)^.

Como outro exemplo, podemos citar a *Multinational Association of Supportive Care in Cancer / International Society of Oral Oncology* (MASCC/ISOO), que, em 2019, forneceu recomendações atualizadas para a prevenção e tratamento da mucosite oral em pacientes com câncer, devido à sua alta recorrência causada por tratamentos como quimioterapia e radioterapia. Nesse sentido, abordar estratégias para a redução da incidência e da gravidade da mucosite é essencial para a prática do manejo ao paciente, por incluir o uso de cuidados orais apropriados, técnicas de manejo da dor e intervenções farmacológicas, com ênfase na abordagem multidisciplinar do cuidado, no intuito de melhorar a qualidade de vida dos pacientes e otimizar os resultados dos tratamentos oncológicos^(32)^.

### Limitações do Estudo

Como possíveis limitações, destaca-se a inclusão apenas de estudos disponíveis publicamente na íntegra, o que pode ter ocasionado a perda de estudos relevantes que não se adequavam a esse critério. Além disso, observa-se o baixo nível de evidência (III e IV) das publicações elencadas para a amostra e sua fragilidade metodológica.

### Contribuições para a Área da Enfermagem, Saúde ou Política Pública

Este estudo oferece contribuições para a compreensão das complexidades envolvidas na assistência a pacientes com neoplasias hematológicas, ao mesmo tempo em que aponta áreas que requerem melhorias, como a formação contínua dos profissionais e a ampliação do acesso aos serviços de saúde. Portanto, novas pesquisas devem concentrar-se no desenvolvimento de estratégias para superar as barreiras identificadas, além de explorar tecnologias e abordagens inovadoras que possam otimizar o cuidado a esses pacientes.

## CONSIDERAÇÕES FINAIS

No que diz respeito aos fatores encontrados nos contextos analisados acerca da assistência a pacientes onco-hematológicos nos serviços de saúde, destacam-se as interações complexas que vão desde necessidades técnicas e interpessoais até desafios relacionados à infraestrutura e à comunicação.

Entre os principais achados, salienta-se a relevância do planejamento antecipado de cuidados, a necessidade de capacitação contínua dos profissionais, bem como a utilização de tecnologias em saúde para aprimorar a qualidade do atendimento. Entretanto, identificou-se a falta de treinamento formal da equipe de saúde e as dificuldades de acesso aos serviços para pacientes em áreas remotas, fatores que podem comprometer a eficácia do cuidado.

Assim, torna-se pertinente a realização e divulgação de outras pesquisas sobre o tema com base em experiências da prática assistencial, além da necessidade de elaboração de estratégias para fortalecer a assistência à saúde do paciente onco-hematológico.

## Data Availability

Não informado o uso de dados, não utilizou dados de pesquisa.
